# The Uncertain Effect of Antimicrobial Therapy in the Treatment of Patients with Ischemic Colitis

**DOI:** 10.3390/jcm9072182

**Published:** 2020-07-10

**Authors:** Jae Gon Lee, Jin Hwa Park, Dong Soo Han, Hang Lak Lee, Chan Hyuk Park, Chang Soo Eun

**Affiliations:** 1 Department of Internal Medicine, Hanyang University Guri Hospital, Hanyang University College of Medicine, Guri 11923, Korea; jaegonlee52@gmail.com (J.G.L.); yesable7@gmail.com (C.H.P.); cseun@hanyang.ac.kr (C.S.E.); 2 Department of Internal Medicine, Hanyang University Hospital, Hanyang University College of Medicine, Seoul 04763, Korea; pjh6718@hanmail.net

**Keywords:** ischemic colitis, anti-bacterial agents, treatment outcome, prognosis

## Abstract

Although antimicrobial therapy is recommended for patients with moderate or severe ischemic colitis, its beneficial effects are unclear. In the present study, the role of antimicrobial therapy in the treatment of ischemic colitis was investigated. Patients with ischemic colitis were retrospectively identified between January 2004 and June 2019. The characteristics and outcomes of patients who received antibiotics (antibiotics group) and those who did not (no-antibiotics group) were compared. Clinical outcomes included death, surgery, and readmission within 30 days, fasting duration, and hospital stay. Data from 186 patients were analyzed; 122 patients were in the antibiotics group and 64 in the no-antibiotics group. Composite outcome of death, surgery, and readmission within 30 days occurred in 3.3% of patients in the antibiotics group and 3.1% of patients in the no-antibiotics group (*p* > 0.999). Fasting duration was not significantly different between the two groups (median days, 4.0 vs. 4.0, *p* = 0.253). However, hospital stays were longer in the antibiotics group than in the no-antibiotics group (median days, 9.0 vs. 7.0, *p* = 0.043). In patients with ischemic colitis, there was no statistically significant difference in the incidence of death, surgery, and readmission within 30 days between patients who received antibiotics and those who did not receive antibiotics.

## 1. Introduction

Ischemic colitis is the most common form of gastrointestinal ischemia, which results from transient alterations of the mesenteric blood flow [1,2]. The incidence of ischemic colitis is reportedly 16.3 cases per 100,000 person-years in a recent population-based study and comprises the major cause of hospitalization for acute lower gastrointestinal bleeding [3,4].

Ischemic colitis involves a wide variety of clinical spectra, ranging from transient or reversible ischemia that resolves spontaneously to fulminant colitis that may cause death [2]. Accordingly, treatment for ischemic colitis varies with the severity of the disease. The majority of patients with ischemic colitis improve with only conservative treatment, including bowel rest, intravenous hydration, and correction of electrolyte imbalance [2]. Conversely, patients with peritoneal signs, massive bleeding, or fulminant colitis require surgical treatment, leading to high morbidity and mortality [5].

Clinical guidelines for the diagnosis and management of ischemic colitis by the American College of Gastroenterology (ACG) provide a disease severity classification system and treatment strategies based on disease severity [2]. Patients with mild disease can expect to recover with supportive care alone, whereas surgical intervention should be considered in patients with severe disease. In addition, antimicrobial therapy with broad-spectrum antibiotics should be considered for patients with moderate or severe disease [2]. Antimicrobial therapy has been reported to attenuate reperfusion injury and improve survival in experimental models of ischemic colitis [6,7]. However, because clinical trials on the efficacy of antimicrobial therapy in the treatment of ischemic colitis have not been performed, it is unclear if the administration of broad-spectrum antibiotics has a beneficial effect in clinical practice. Therefore, we conducted a study to investigate the role of broad-spectrum antibiotics in the treatment of ischemic colitis.

## 2. Materials and Methods

### 2.1. Patients

Patients with ischemic colitis were retrospectively identified at two university-affiliated hospitals between 1 January, 2004 and 30 June, 2019. Patients first diagnosed with and hospitalized for ischemic colitis were eligible for inclusion in the study. Diagnosis of ischemic colitis was reaffirmed when the clinical and endoscopic findings were consistent with ischemic colitis and the stool culture was negative for enteric pathogens including enterohemorrhagic *Escherichia coli*, *Clostridioides difficile* (*C. difficile*), and cytomegalovirus. Clinical findings for the diagnosis of ischemic colitis included sudden abdominal pain and/or rectal bleeding, and endoscopic findings included segmental colitis with linear mucosal edema, erythema, petechiae, or ulcerations. Patients with active systemic infection, advanced colorectal cancer, metastatic carcinoma, history of colectomy, intraabdominal vascular disease, and peritoneal signs at presentation were excluded.

### 2.2. Data Collection and Definition

Medical records were reviewed to collect patients’ demographic and clinical data. Blood pressure, pulse rate, presence of abdominal pain, rectal bleeding, nausea and/or vomiting, diarrhea, and fever at presentation were identified. The presence of comorbidities such as hypertension, diabetes, cardiovascular disease, chronic kidney disease, and chronic pulmonary disease was also investigated. Patients were divided into two groups depending on whether antibiotics were administered or not, the antibiotics group and the no-antibiotics group.

Laboratory data, including complete blood count, electrolytes, and chemistry panels were collected. Abdominal computed tomography (CT) findings were reviewed to identify the presence of colonic wall thickening and/or edema, pericolic infiltration, and decrease in contrast enhancement. Colonoscopy findings were reviewed to identify the presence of bowel edema, erythema, petechiae, ulcer, and friability. The ulcers were divided into superficial ulcers and deep ulcers by visual assessment of the investigators; a superficial ulcer was defined as an ulcer with a depth of approximately <3 mm, and a deep ulcer was defined as an ulcer with a depth of approximately ≥3 mm. In addition, blood culture results were reviewed to identify patients with bacteremia.

Distribution of the disease was defined as follows: left-sided, disease limited from the splenic flexure to the rectum; right-sided, disease limited from the cecum to the distal transverse colon; bilateral, disease involving both sides of the colon. Distribution was determined based on the colonoscopy findings if total colonoscopy was performed, and was determined based on the CT findings if only sigmoidoscopy was performed.

Severity of the disease was classified as mild, moderate, or severe according to the criteria proposed in the ACG guidelines [2]. Mild disease was considered a segmental colitis that was not isolated to the right colon and without risk factors associated with poor outcomes observed in moderate disease. Moderate disease included patients with up to three of the following risk factors associated with poor outcomes: male sex, hypotension (systolic blood pressure < 90 mmHg), tachycardia (heart rate > 100 beats/min), abdominal pain without rectal bleeding, blood urea nitrogen > 20 mg/dL, hemoglobin < 12 g/dL, lactate dehydrogenase (LDH) > 350 U/L, serum sodium < 136 mmol/L, white blood cell (WBC) > 15,000 per µL, or colonoscopically identified mucosal ulceration. Severe disease included patients with peritoneal signs, pneumatosis on CT, gangrene on colonoscopy, pancolonic or isolated right colon ischemia, or having more than three risk factors associated with poor outcomes.

Clinical outcomes included all-cause mortality, surgery, and readmission within 30 days, fasting duration, and hospital stay. To assess response to treatment, changes in WBC counts over time were measured and compared between groups.

### 2.3. Ethical Approval

The study protocol conformed to ethical guidelines of the 1975 Declaration of Helsinki and was approved by the Institutional Review Board (IRB) on Human Subjects Research and Ethics Committees of Hanyang University Guri Hospital (IRB No. 2019-08-020) and Hanyang University Hospital (IRB No. 2019-08-025). Informed consent was waived by the board because the study was conducted retrospectively with de-identified data.

### 2.4. Statistical Analysis

Continuous variables were expressed as mean ± standard deviation or median (interquartile range, IQR). Categorical variables were expressed as numbers (with proportions). For comparison of the two groups, the *t*-test or Mann–Whitney test were used for continuous variables and the chi-square test with Fisher’s exact test for categorical variables. Logistic regression analysis was performed to determine the factors associated with the composite outcome of death, surgery, and readmission within 30 days. Linear regression analysis was performed to determine the factors associated with length of hospital stay. A *p*-value <0.05 was considered statistically significant. All statistical analyses were performed using R statistical language RStudio version 3.4.3 (R Foundation for Statistical Computing, Vienna, Austria).

## 3. Results

### 3.1. Baseline Characteristics

**Figure 1:**
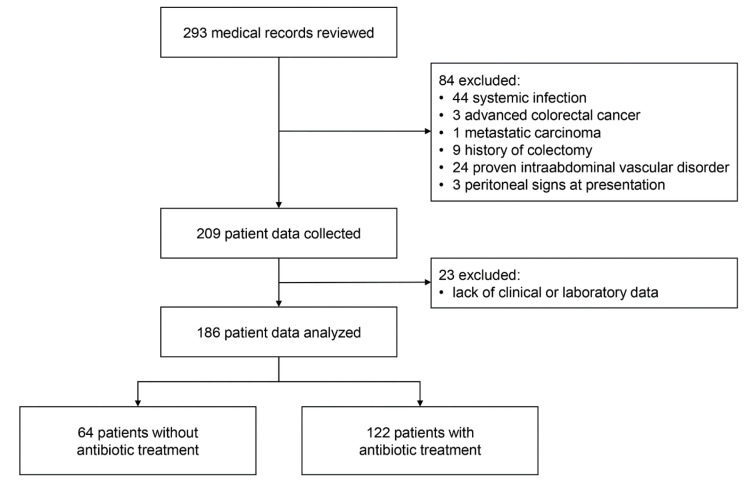
Study flow diagram.

A total of 293 patients with ischemic colitis were identified and their medical records were reviewed; 44 patients with systemic infection, 3 with advanced colorectal cancer, 1 with metastatic carcinoma, 9 with history of colectomy, 24 with intraabdominal vascular disorder, and 3 with peritoneal signs at presentation were excluded. After exclusion, clinical data for 209 patients were collected. In addition, 23 patients lacking clinical or laboratory data were excluded. Finally, data of 186 patients were analyzed (Figure 1).

Table 1 shows the baseline characteristics of the patients. Among the 186 patients, the antibiotics group included 122 patients and the no-antibiotics group included 64 patients. Types of antibiotics administered were 54.9% for 3rd generation cephalosporins or fluoroquinolones, 39.3% for metronidazole plus 3rd generation cephalosporins or fluoroquinolones, and 5.7% for piperacillin/tazobactam. Duration of antibiotic treatment was a median of 7 days (IQR, 5–9 days).

Among the patients, 74.2% were female and the median age was 69.0 years. The most common clinical symptoms were abdominal pain and bloody stools (88.7% and 89.2%, respectively). The urge to defecate was present in 30.3% of patients in the antibiotics group and 15.6% in the no-antibiotics group (*p* = 0.044). Patients’ vital signs and comorbidities were not significantly different between the two groups.

Based on laboratory results, WBC counts were significantly higher in the antibiotics group than in the no-antibiotics group (median WBC count, 11,350 per µL vs. 9900 per µL, *p* = 0.013). Absolute neutrophil counts were also higher in the antibiotics group than in the no-antibiotics group (median absolute neutrophil count, 8594.8 per µL vs. 7659.6 per µL, *p* = 0.017). Serum albumin and LDH levels were significantly lower in the antibiotics group than in the no-antibiotics group (median albumin level, 3.9 g/dL vs. 4.2 g/dL, *p* = 0.012; median LDH level, 260.5 U/L vs. 426.5 U/L, *p* < 0.001). Blood cultures were performed before antibiotic administration in 96 of the 122 patients (78.7%) in the antibiotics group and no patients were identified as having bacteremia.

Abdominal CT was performed in 172 of 186 patients (92.5%). Bowel wall thickening and/or edema was the most common finding (86.6%) and was 91.2% in the antibiotics group and 77.6% in the no-antibiotics group (*p* = 0.025). Pericolic infiltration was present in 48.8% of patients.

Colonoscopy was performed in all included patients on median 1 day (IQR, 0–2 days) after presentation; the most common findings were bowel edema and erythema, both were present in 98.9% of patients. Colonic ulcerations were identified in 77.9% of patients; 29.0% superficial ulcers and 48.9% deep ulcers. Mucosal friability was observed in 41.0% of patients in the antibiotics group and 25.0% in the no-antibiotics group (*p* = 0.045). Colonoscopic biopsy was performed in 144 of 186 patients (77.4%); 108 of 144 patients (75%) were confirmed histologically consistent with ischemic colitis.

### 3.2. Clinical Outcomes

Table 2 shows the major clinical outcomes. Composite outcome of death, surgery, and readmission within 30 days occurred in 3.3% of patients in the antibiotics group and 3.1% in the no-antibiotics group (*p* > 0.999). Fasting duration did not differ significantly between the antibiotics and no-antibiotics groups. However, the length of hospital stay was significantly longer in the antibiotics group than in the no-antibiotics group.

In the subgroup of patients with moderate disease, composite outcome occurred in 2 of 79 patients who received antibiotics and 0 of 46 patients who did not receive antibiotics (2.5% vs. 0%, *p* = 0.727). There was no significant difference in fasting duration between the patients who received antibiotics and those who did not (median days, 4.0 vs. 4.0, *p* = 0.458), but the length of hospital stay was significantly longer in patients who received antibiotics than in those who did not (median days, 9.0 vs. 7.0, *p* = 0.043). In the severe disease subgroup, composite outcome occurred in 1 of 23 patients who received antibiotics and 2 of 12 patients who did not receive antibiotics (4.3% vs. 16.7%, *p* = 0.549). Fasting duration and hospital stay did not differ significantly between patients who received antibiotics and those who did not.

In a subgroup analysis according to the depth of colonic ulcers, no death, surgery, or readmission within 30 days occurred in 54 patients with superficial ulcers. Among 91 patients with deep ulcers, composite outcome occurred in 2 of 61 patients who received antibiotics and 2 of 30 patients who did not receive antibiotics (3.3% vs. 6.7%, *p* = 0.844).

**Figure 2:**
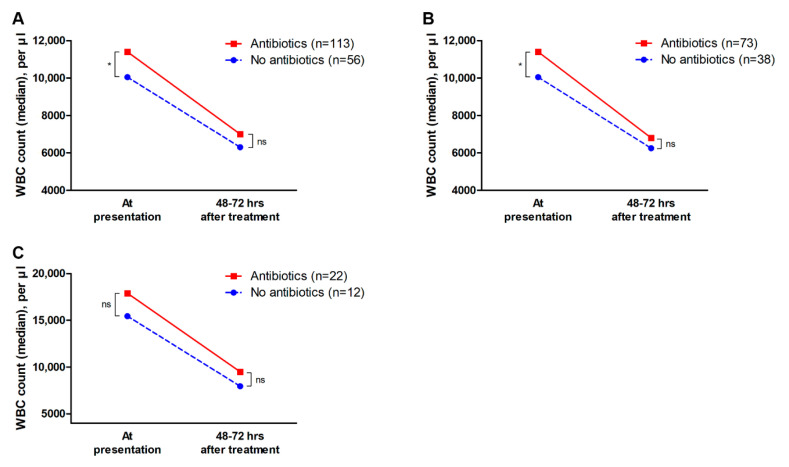
Changes in white blood cell counts. Data of 169 patients who had follow-up WBC counts at 48–72 h after hospitalization were compared. (**A**) In all patients with ischemic colitis, the antibiotics group had significantly higher WBC counts at presentation than the no-antibiotics group, however, WBC counts at 48–72 h after hospitalization did not significantly differ between the two groups. (**B**) In the subgroup of patients with moderate disease, changes in WBC counts were similar to those in the entire patient population. (**C**) In the subgroup of patients with severe disease, WBC counts were not significantly different between the two groups both at presentation and after 48–72 h of treatment. WBC, white blood cell. * *p* < 0.05; ns, non-significant.

In addition, changes in the WBC counts of 169 patients who had follow-up WBC counts at 48–72 h after hospitalization were analyzed; the median WBC counts at presentation were 11,400 per µL and 10,500 per µL in the antibiotics and no-antibiotics groups, respectively (*p* = 0.019). However, after 48–72 h of hospitalization, WBC count did not significantly differ between the antibiotics and no-antibiotics groups (median WBC count, 7000 per µL vs. 6300 per µL, *p* = 0.148). These results were similar in the moderate and severe disease subgroups (Figure 2). In addition, among the 108 patients with leukocytosis (WBC counts ≥ 10,000 per µL) at presentation, 22 patients in the antibiotics group (27.2%, 22 of 81 patients) and 6 patients in the no-antibiotics group (22.2%, 6 of 27 patients) had persistent leukocytosis after 48–72 h of hospitalization (*p* = 0.801).

### 3.3. Factors Associated with Outcomes

The logistic regression analysis of the factors associated with the composite outcome of death, surgery, and readmission within 30 days is shown in Table 3. The major variables including age, sex, use of antibiotics, number of risk factors, disease distribution, presence of ulcers, and disease severity were not significantly associated with the composite outcome of death, surgery, and readmission within 30 days.

Factors associated with length of hospital stay based on a simple linear regression analysis included age, number of risk factors, bilateral disease, presence of deep ulcer, and severe disease. In the multiple linear regression analysis, disease severity was associated with length of hospital stay. Use of antibiotics was not associated with the length of hospital stay (Table 4).

### 3.4. Adverse Events Associated with Antibiotic Treatment

Among the 122 patients who received antibiotic treatment, nausea/vomiting occurred in 5 patients (4.1%), non-*C. difficile*-associated diarrhea in 2 patients (1.6%), dermatologic abnormalities in 2 patients (1.6%), hematologic abnormalities in 12 patients (9.8%), hepatotoxicity in 1 patient (0.8%), and renal impairment in 1 patient (0.8%). *C. difficile* infection did not occur in any patient.

### 3.5. Causes of Death, Surgery, and Readmission

Death within 30 days occurred in one patient in the antibiotics group and one in the no-antibiotics group; both were identified as cardiac deaths. One patient in the antibiotics group underwent colectomy for fulminant colitis and survived after surgery without postoperative complications (Supplementary Table S1).

## 4. Discussion

In the present study, the beneficial effects of broad-spectrum antibiotics in the treatment of ischemic colitis were found to be uncertain. There was no statistically significant difference in the incidence of death, surgery, and readmission within 30 days between patients who received antibiotics and those who did not. The length of hospital stay was longer in patients who received antibiotics than in those who did not. The number of risk factors and disease distribution were identified as factors associated with the length of hospital stay.

We confirmed that patients with ischemic colitis who had no acute indication of surgery at presentation had an excellent prognosis. In previous literature, the mortality rates in patients with ischemic colitis ranged from 4%–12% and surgery rates from 9%–20% [3,8,9,10,11]. In the present study, death occurred in 2 of 186 patients (1.1%) and colectomy was required in 1 of 186 patients (0.5%). The low mortality and surgery rates in the present study are presumably because patients with acute peritoneal signs at presentation were excluded. The results support that non-occlusive, reversible colon ischemia has an excellent prognosis.

Indications for antimicrobial therapy in patients with ischemic colitis have not been investigated in clinical trials. Although the guidelines recommend that antibiotic treatment be considered for moderate or severe disease [2], antibiotic treatment is likely determined by a physician’s judgment in real-life clinical practice. A systematic review of the treatment for ischemic colitis indicated that there are no established protocols for the treatment of patients with ischemic colitis [12]. Surgery for patients with peritonitis is undoubtedly accepted, however, a consensus on medical treatment is lacking. Meanwhile, in previous studies on prognostic factors of ischemic colitis, antibiotic therapy was reported as a factor associated with colectomy or death [9,13]. However, these findings may indicate that antimicrobial therapy was administered to the most severely ill patients who are at the highest risk of poor outcomes regardless of medical therapy. In the present study, the WBC count in the antibiotics group was higher than in the no-antibiotics group, suggesting that antibiotics tend to be given to patients with more severe disease. Notably, clinical outcomes were not statistically significantly different between the antibiotics and no-antibiotics groups, even though antibiotics tended to be administered to patients with more severe disease. On the other hand, these results also suggest that patients with more severe disease might benefit from antimicrobial therapy and have favorable outcomes similar to patients who did not receive antimicrobial therapy.

Evidence for antimicrobial therapy in patients with ischemic colitis is based on experimental studies. In a murine colon ischemia model in which the superior mesenteric artery was ligated for 30 min and then reperfused, mice treated with broad-spectrum antibiotics showed a decreased expression of inflammatory markers and inhibited complement activation, resulting in attenuation of intestinal inflammation associated with ischemia [6]. In addition, intestinal ischemia and reperfusion injury trigger gut bacterial translocation [14]. Broad-spectrum antibiotics are believed to reduce bacterial translocation by depleting gut bacteria. However, the efficacy and safety of broad-spectrum antibiotics in the treatment of patients with ischemic colitis has not been investigated in human studies. Although animal studies have shown possible therapeutic benefits of antimicrobial therapy in intestinal ischemia models, applying those results to human patients may be inappropriate. First, unlike the experimental model, the majority of colon ischemia in humans is transient, reversible ischemia [2,15]. Irreversible transmural infarction rarely occurs and patients with irreversible infarction usually show acute indications for surgery at presentation [2,15]. In the present study, among patients with ischemic colitis who had no acute surgical indications at presentation, statistically significant differences in mortality and surgery rates were not observed between patients who received antibiotics and those who did not. Reductions in WBC counts were also similar between the antibiotics and no-antibiotics groups. The results suggest that antimicrobial therapy may not significantly affect disease course, and patients without acute surgical indications have favorable outcomes regardless of antimicrobial therapy. In addition, ischemic colitis is not an infectious disease, and there is no evidence that bacterial translocation results in clinically significant bacteremia. In the present study, bacteremia confirmed by blood cultures did not occur in any patient.

The length of hospital stay in the antibiotics group was longer than in the no-antibiotics group. This may raise concerns that antimicrobial therapy increases the length of hospital stay. However, the disease severity, not the use of antibiotics, was associated with the length of hospital stay based on multiple regression analysis. These results suggest that the treatment duration is determined by disease severity. Therefore, patients with severe disease will be more likely to receive antimicrobial therapy and increase their length of hospital stay accordingly.

The present study results call for reconsideration of the routine use of antibiotics for all patients with ischemic colitis. The use of antibiotics may cause potential toxicity, increased medical costs, emergence of antibiotic-resistant bacteria, and *C. difficile* infection [16,17]. Therefore, antibiotics should only be used when the benefits of their use are evident. Although the results of this study showed that there was no statistically significant difference in mortality, need for surgery, and readmission within 30 days between patients who received antibiotics and those who did not, we cannot conclude that antimicrobial therapy is unnecessary for patients with ischemic colitis. A prospective randomized controlled study is needed to answer this issue.

This study had several limitations. First, because the data were collected retrospectively, some variables were not standardized and the depth of colonic ulcers was measured by the investigators’ visual assessment. In addition, the 2015 ACG guidelines were applied to patients included before 2015 for classification of disease severity. Moreover, the retrospective study design precludes determination of a causal relationship between antimicrobial therapy and clinical outcomes. However, as the results showed, the prognosis of ischemic colitis was excellent in most cases. Therefore, a prospective randomized controlled trial is difficult to conduct because the number of samples needed to demonstrate the benefits of antimicrobial therapy would be extremely large. The prominent clinical implication of this study is that antimicrobial therapy for ischemic colitis may not have a significant benefit, especially in patients without acute surgical indications. Second, because we excluded patients with peritoneal signs at presentation, selection bias may have been introduced. Consequently, both mortality and surgery rates were lower compared with previous literature. In addition, the results of this study cannot be generalized to the management of patients with surgical indications. However, by excluding patients with peritoneal signs, we could investigate the clinical outcomes of patients who were determined to undergo medical treatment at the initial assessment. The results indicated that patients without acute surgical indications have an excellent prognosis regardless of antimicrobial therapy. Third, antimicrobial therapy was determined by physician’s clinical judgement without definite indications. This is because many patients before the publication of the guidelines were included, and the level of evidence for antimicrobial therapy was low in the guidelines.

Despite these limitations, the results of this study provide a better understanding of the role of antimicrobial therapy in the treatment of patients with ischemic colitis. In patients with ischemic colitis who do not have acute surgical indications, there was no statistically significant difference in the incidence of death, surgery, and readmission within 30 days between patients who received antibiotics and those who did not.

## Figures and Tables

**Table 1:** Baseline characteristics.

Variable	Antibiotics (*n* = 122)	No Antibiotics (*n* = 64)	*p*-Value
Age, years	70.5 (60.0–79.0)	67.0 (54.5–76.0)	0.083
Sex			0.287
Female	87 (71.3)	51 (79.7)	
Male	35 (28.7)	13 (20.3)	
Clinical features			
Abdominal pain	111 (91.0)	54 (84.4)	0.267
Rectal bleeding	107 (87.7)	59 (92.2)	0.491
Diarrhea	63 (51.6)	30 (46.9)	0.643
Nausea and/or vomiting	13 (10.7)	14 (21.9)	0.065
Urge to defecate	37 (30.3)	10 (15.6)	0.044
Fever	5 (4.1)	4 (6.2)	0.772
Vital signs			
Systolic BP, mmHg	122.5 (110.0–140.0)	120.0 (110.0–140.0)	0.776
Heart rate, beats/min	78.0 (70.0–84.0)	78.0 (72.0–88.0)	0.430
Comorbidities			
Hypertension	65 (53.3)	34 (53.1)	>0.999
Diabetes mellitus	29 (23.8)	15 (23.4)	>0.999
Atrial fibrillation	4 (3.3)	2 (3.1)	>0.999
Congestive heart failure	4 (3.3)	4 (6.2)	0.570
Ischemic heart disease	12 (9.8)	9 (14.1)	0.534
Cerebrovascular disease	19 (15.6)	5 (7.8)	0.204
Peripheral artery disease	2 (1.6)	0 (0.0)	0.778
Chronic pulmonary disease	6 (4.9)	3 (4.7)	>0.999
Chronic kidney disease	14 (11.5)	6 (9.4)	0.849
Constipation	19 (15.6)	7 (10.9)	0.520
Non-metastatic cancer	3 (2.5)	4 (6.2)	0.376
Laboratory findings			
WBC count, per µL	11,350.0 (9500.0–14,300.0)	9900.0 (7950.0–13,300.0)	0.013
Neutrophil count, per µL	8594.8 (6814.8–11,816.4)	7659.6 (5845.8–10,448.9)	0.017
Hemoglobin, g/dL	13.4 (12.4–14.2)	13.0 (11.6–14.1)	0.092
Platelet count, ×10^9^/L	214.5 (182.0–247.0)	212.0 (170.5–276.0)	0.781
Sodium, mmol/L	139.0 (137.0–141.0)	139.0 (136.5–140.5)	0.901
Potassium, mmol/L	3.8 (3.6–4.1)	3.8 (3.6–4.1)	0.398
Albumin, g/dL	3.9 (3.6–4.2)	4.2 (3.8–4.4)	0.012
BUN, mg/dL	17.1 (12.5–24.5)	15.0 (10.9–19.5)	0.081
Creatinine, mg/dL	0.8 (0.6–1.0)	0.8 (0.6–1.0)	0.886
Amylase, U/L	51.5 (42.0–71.0)	54.0 (44.0–75.0)	0.487
LDH, U/L	260.5 (199.0–403.0)	426.5 (335.0–507.5)	<0.001
Creatine kinase, U/L	75.0 (55.0–135.0)	74.0 (49.0–112.0)	0.461
CRP, mg/dL	2.2 (0.4–6.2)	1.5 (0.5–3.3)	0.366
CT findings *			
Bowel wall thickening and/or edema	104 (91.2)	45 (77.6)	0.025
Pericolic Infiltration	60 (52.6)	24 (41.4)	0.217
Decreased contrast enhancement	20 (17.5)	4 (6.9)	0.094
Colonoscopy findings			
Edema	122 (100.0)	62 (96.9)	0.224
Erythema	121 (99.2)	63 (98.4)	>0.999
Petechiae	89 (73.0)	45 (70.3)	0.834
Superficial ulcer	39 (32.0)	15 (23.4)	0.295
Deep ulcer	61 (50.0)	30 (46.9)	0.802
Friability	50 (41.0)	16 (25.0)	0.045
Distribution			0.708
Left-sided	95 (77.9)	53 (82.8)	
Right-sided	4 (3.3)	2 (3.1)	
Bilateral	23 (18.9)	9 (14.1)	
Disease severity			0.407
Mild	20 (16.4)	6 (9.4)	
Moderate	79 (64.8)	46 (71.9)	
Severe	23 (18.9)	12 (18.8)	

Categorical and continuous variables are presented as numbers (%) and medians (interquartile range), respectively. BP, blood pressure; WBC, white blood cell; BUN, blood urea nitrogen; LDH, lactate dehydrogenase; CRP, C-reactive protein; CT, computed tomography. * Abdominal CT was performed in 172 of 186 patients (92.5%); 58 of the 172 patients were not treated with antibiotics and 114 were treated with antibiotics.

**Table 2:** Clinical outcomes.

Variable	Antibiotics (*n* = 122)	No Antibiotics (*n* = 64)	*p*-Value
Composite outcome	4 (3.3)	2 (3.1)	>0.999
Death within 30 days	1 (0.8)	1 (1.6)	>0.999
Surgery within 30 days	1 (0.8)	0 (0.0)	>0.999
Readmission within 30 days	2 (1.6)	1 (1.6)	>0.999
Fasting duration, days	4.0 (3.0–6.0)	4.0 (3.0–5.0)	0.117
Hospital stay, days	9.0 (6.0–11.0)	7.0 (5.5–9.5)	0.043

Categorical and continuous variables are presented as numbers (%) and medians (interquartile range), respectively.

**Table 3:** Factors associated with composite outcome of death, surgery, and readmission within 30 days.

Variable	Univariable Analysis		Multivariable Analysis *	
	OR (95% CI)	*p*-Value	OR (95% CI)	*p*-Value
Age	1.039 (0.972–1.109)	0.261	1.035 (0.965–1.111)	0.336
Number of risk factors ^†^	1.415 (0.851–2.353)	0.181		
Sex				
Female	1.000			
Male	3.000 (0.585–15.396)	0.188		
Use of antibiotics				
No antibiotics	1.000		1.000	
Antibiotics	1.051 (0.187–5.898)	0.955	0.926 (0.157–5.458)	0.932
Disease distribution				
Left-sided	1.000			
Right-sided	5.720 (0.559–58.488)	0.141		
Bilateral	0.000 (0.000)	0.998		
Presence of deep ulcer				
No	1.000			
Yes	2.138 (0.382–11.968)	0.387		
Disease severity				
Mild	1.000		1.000	
Moderate	0.407 (0.035–4.658)	0.469	0.329 (0.027–3.963)	0.382
Severe	2.344 (0.230–23.917)	0.472	1.649 (0.147–18.454)	0.685

OR, odds ratio; CI, confidence interval. * Multivariable analysis included age, use of antibiotics, and disease severity. ^†^ Risk factors include male sex, hypotension (systolic BP < 90 mmHg), tachycardia (heart rate > 100 beats/min), abdominal pain without rectal bleeding, BUN > 20 mg/dL, hemoglobin < 12 g/dL, LDH > 350 U/L, serum sodium < 136 mmol/L, WBC > 15,000 per µL.

**Table 4:** Factors associated with length of hospital stay.

Variable	Univariable Analysis		Multivariable Analysis *	
	B (95% CI)	*p*-Value	B (95% CI)	*p*-Value
Age	0.068 (0.019–0.116)	0.006	0.049 (−0.001–0.099)	0.054
Male sex	−0.475 (−2.163–1.214)	0.580		
Use of antibiotics	1.228 (−0.318–2.774)	0.119	1.166 (−0.367–2.699)	0.135
Number of risk factors ^†^	0.932 (0.452–1.411)	<0.001		
Right-sided disease	2.266 (−1.853–6.385)	0.279		
Bilateral disease	2.682 (0.754–4.611)	0.007		
Presence of deep ulcer	2.016 (0.566–3.466)	0.007		
Moderate disease	2.082 (−0.055–4.219)	0.056	1.906 (−0.245–4.057)	0.082
Severe disease	3.533 (0.966–6.100)	0.007	3.038 (0.415–5.661)	0.023

B, unstandardized regression coefficient; CI, confidence interval. * Multivariable analysis included age, use of antibiotics, and disease severity. ^†^ Risk factors include male sex, hypotension (systolic BP < 90 mmHg), tachycardia (heart rate > 100 beats/min), abdominal pain without rectal bleeding, BUN > 20 mg/dL, hemoglobin < 12 g/dL, LDH > 350 U/L, serum sodium < 136 mmol/L, WBC > 15,000 per µL.

## Supplementary Materials

The following are available online at https://www.mdpi.com/2077-0383/9/7/2182/s1, Table S1: Characteristics of patients in whom the composite outcome of death, surgery, and readmission within 30 days occurred.
