# Supplementary material for: The Uncertain Effect of Antimicrobial Therapy in the Treatment of Patients with Ischemic Colitis
Source: J Clin Med. 2020 Jul 10;9(7):2182. doi: 10.3390/jcm9072182 (PMC7408655; doi:10.3390/jcm9072182)
Supplement: Supplementary file 1 [file jcm-09-02182-s001.pdf]

Supplementary Materials

Table S1. Characteristics of patients in whom the composite outcome of death, surgery, and readmission within 30 days occurred.

Variable	Patient					
	A	B	C	D	E	F
Age (years)	83	80	67	68	63	83
Sex	Female	Male	Male	Female	Male	Female
Use of antibiotics	Yes	Yes	No	Yes	Yes	No
Abdominal pain without bleeding	No	No	No	Yes	Yes	No
Systolic BP (mmHg)	130	130	140	102	123	130
Heart rate (beats/min)	100	84	114	72	57	78
Comorbidities	None	HTN	HTN, DM, IHD, CKD	HTN, CKD	HTN	DM, AF, CKD
WBC count (per μL)	9,900	13,500	15,100	10,100	8,000	5,900
Hemoglobin (g/dL)	12.3	12.5	9.3	12.2	14.3	8.1
Sodium (mmol/L)	139	138	136	139	140	133
BUN (mg/dL)	14.8	19.1	72	46	8	38
LDH (U/L)	243	260	514	535	284	426
Presence of colonic ulcer	No	Yes	Yes	No	Yes	Yes
Distribution	Left-sided	Left-sided	Left-sided	Left-sided	Right-sided	Left-sided
Number of risk factors*	0	1	6	3	2	4
Disease severity	Mild	Moderate	Severe	Moderate	Moderate	Severe
Causes of outcome	Readmission due to recurrence of abdominal pain	Surgery due to fulminant colitis	Death due to myocardial infarction	Death due to cardiac arrest	Readmission due to recurrence of abdominal pain	Readmission due to recurrence of bleeding

BP, blood pressure; WBC, white blood cell; BUN, blood urea nitrogen; LDH, lactate dehydrogenase; HTN, hypertension; DM, diabetes mellitus; IHD, ischemic heart disease; CKD, chronic kidney disease; AF, atrial fibrillation. *Risk factors include male sex, hypotension (systolic BP <90 mmHg), tachycardia (heart rate >100 beats/min), abdominal pain without rectal bleeding, BUN >20 mg/dL, hemoglobin <12 g/dL, LDH >350 U/L, serum sodium <136 mmol/L, WBC >15,000 per μL.
